# Use of Two Biologics During Pregnancy: Infliximab and Vedolizumab: A Case Study

**DOI:** 10.1097/FTD.0000000000001519

**Published:** 2026-09-23

**Authors:** Birgit Hoekstra, Dianne G. Bouwknegt, Paola Mian, Gerard Dijkstra, Martha K. Leijsma, Mariëtte Groenewout, Marijn C. Visschedijk

**Affiliations:** * Department of Gastroenterology and Hepatology, University Medical Center Groningen, University of Groningen, Groningen, the Netherlands;; † Department of Clinical Pharmacy and Pharmacology, University Medical Center Groningen, University of Groningen, Groningen, the Netherlands;; ‡ Department of Pediatrics, Beatrix Children's Hospital, University Medical Center Groningen, University of Groningen, Groningen, the Netherlands;; § Department of Rheumatology and Clinical Immunology, University Medical Center Groningen, University of Groningen, Groningen, the Netherlands; and; ¶ Department of Gynaecology and Obstetrics, University Medical Center Groningen, University of Groningen, Groningen, the Netherlands.

**Keywords:** therapeutic drug monitoring, inflammatory bowel disease, pregnancy, dual biologic treatment

## Abstract

**Background:**

Pregnancy is associated with altered pharmacokinetics for several drugs.

The blood concentration of infliximab increases during pregnancy, whereas that of vedolizumab tends to decrease. As autoimmune disease activity during pregnancy can lead to adverse pregnancy outcomes, monitoring drug concentrations during pregnancy is important.

**Methods:**

This study reports a 32-year-old female patient with severe juvenile idiopathic arthritis and Crohn disease who was treated with dual biologic therapy throughout her pregnancy. The measurements were performed as part of routine care.

**Results:**

The patient was treated with vedolizumab and infliximab during the course of her pregnancy. Both autoimmune diseases remained unaffected throughout pregnancy, and therapeutic drug concentrations were monitored to guide treatment. The patient had gestational diabetes and mild polyhydramnios. Ultimately, the pregnancy was complicated by preterm delivery at 33 weeks and 5 days of gestation, with a good birth weight of 2425 g at p85 without growth retardation.

**Conclusions:**

Therapeutic drug monitoring (TDM) supports optimal dosing and effective disease control, highlighting the importance of individual interpretations. All TDM-initiated dosing changes were based on expert opinions, owing to the lack of validated guidelines on dosing during pregnancy. However, preterm birth could not be avoided and may be influenced by underlying autoimmune diseases, gestational diabetes, and mild polyhydramnios. Multidisciplinary care is essential in pregnant women with autoimmune disorders. Prospective research focusing on drug concentrations, disease activity, and the risk of adverse pregnancy outcomes is needed to understand the safety and implications of dual biologic therapies during pregnancy.

## CLINICIAN

We describe the case of a 32-year-old woman with severe juvenile idiopathic arthritis since infancy, later complicated by Crohn's disease (CD) diagnosed at the age of 8 years. Over approximately 30 years, she received multiple therapies, often discontinued because of nonresponse, loss of response, or side effects.

Arthritis treatment began with sulfasalazine (375 mg twice daily), followed by methotrexate (7.5 mg weekly) and prednisolone (1.5 mg twice daily). Sulfasalazine was discontinued once arthritis stabilized. An exacerbation led to escalation of methotrexate (22.5 mg weekly) and prednisolone (4.5 mg twice daily), plus etanercept (12.5 mg 2-weekly).

She was diagnosed with CD and mesalazine (dose unknown) was initiated. Drug-related pancreatitis lead to the discontinuation of etanercept and mesalazine.

The patient received several biologics to control both diseases, including infliximab (250 mg 6–8-weekly). Arthritis and CD entered remission, after which infliximab was discontinued and azathioprine (100 mg daily) was initiated. Adalimumab (40 mg 2-weekly) was initiated, but azathioprine and adalimumab were discontinued owing to loss of response in CD. Golimumab (50 mg 4-weekly) was started but discontinued because of active CD and antibodies. The treatment of both diseases with a single biologic was still pursued.

Tocilizumab (8 mg/kg every 8 weeks) with leflunomide (20 mg daily) was initiated after discontinuing methotrexate but was stopped due to pancreatic and hepatic test disorders. Tofacitinib (10 mg twice daily) was administered but withdrawn due to loss of response. Ustekinumab (90 mg 8-weekly) was administered but discontinued for partial CD control. Infliximab (5 mg/kg every 6 weeks) and methotrexate (15 mg weekly) were restarted, but terminated because of active CD despite infliximab. A second course of tofacitinib (10 mg twice daily) was ineffective.

Baricitinib (4 mg daily) was initiated, and controlled only the gastrointestinal symptoms. Following multidisciplinary consultation and considering surgical treatment of CD, two biologics were combined: infliximab (4-weekly 10 mg) and vedolizumab (2-weekly 108 mg), combined with methotrexate and prednisone she was already on.

Owing to the patient's desire to conceive, methotrexate was discontinued.

The patient received both vedolizumab and infliximab throughout pregnancy and remained in remission under both conditions. The pregnancy was complicated by gestational diabetes and mild polyhydramnios, both risk factors for preterm delivery.^1^ At 33 weeks and 5 days, she delivered a healthy infant.

Infliximab and vedolizumab are biologic agents widely used to treat inflammatory bowel disease (IBD).^2^ Emerging evidence suggests that dual biologic therapy may be safe for patients with coexisting IBD and rheumatologic conditions, but only limited data exist.^3,4^ The patient had a long history of nonresponse or loss of response to various biologics. Owing to insufficient disease control with infliximab monotherapy, vedolizumab was administered.

## TDM CONSULTANT

There are no specific guidelines for measuring biological drug concentrations during pregnancy. Routine therapeutic drug monitoring (TDM) and proactive dose adjustments are generally not considered essential, because their impact on clinical decision making has not yet been clearly established.^5^ Instead, dosing strategies are typically guided by clinical experience and expert opinion.

In our study, drug trough concentrations were measured as part of routine care to ensure that they were not excessively low or high, providing the physician a reference point for trough concentrations if disease activity increased, without signs of toxicity. Trough concentrations are commonly used in clinical TDM of monoclonal antibodies and provide practical indicators of systemic exposure. Adherence was assessed from patient history and medical record review.

To our knowledge, no published reports have described the concurrent use of these two biologic agents during pregnancy (nonsystematic PubMed search). Reporting this unique clinical situation improves our understanding of how biological drug concentrations may change during pregnancy and the potential consequences on disease activity and drug-related toxicity.

Pregnancy alters the pharmacokinetics of many drugs.^6^ For infliximab, a tumor necrosis factor-alpha (TNF-α) inhibitor, blood concentrations increase. The concentration of vedolizumab, a monoclonal antibody, tends to remain unchanged or decrease.^6,7^ The reason for this trend remains unclear. Several explanations have been proposed, but substantial changes in the pharmacokinetics of monoclonal antibodies are not generally expected given their relatively small volume of distribution.^6^ TDM helps monitor and optimize the doses of these biologics during pregnancy.

## CLINICIAN

Disease activity during pregnancy in patients with IBD has been associated with adverse pregnancy outcomes, including preterm prelabor rupture of membranes, preterm birth, and low birth weight.^8^ Careful monitoring of pregnant patients with IBD is therefore crucial. In this case, the patient was symptom-free during pregnancy. Based on clinical judgment and in agreement with the patient, the gastroenterologist specializing in IBD extended the vedolizumab interval from 2 to 3.5 weeks to reduce overall biologic exposure. Similarly, the absence of arthritis symptoms led the rheumatologist, in agreement with the patient, to reduce the infliximab dose from 10 to 5 mg every 4 weeks, starting in week 18 of the pregnancy. Both diseases remained well controlled throughout pregnancy.

## TDM CONSULTANT

The serum concentrations of vedolizumab and infliximab were measured using an enzyme-linked immunosorbent assay (ELISA) performed by Sanquin Diagnostic Services.^9,10^ In our patient, vedolizumab concentrations declined during pregnancy and increased again postpartum, following the pattern described in the literature (Fig. 1).^6,7^ The interval was increased from 2 to 3.5 weeks around week 19 and changed to 3 weeks postpartum. Concentrations fell from 15 mcg/mL before pregnancy to 5.3 mcg/mL at week 20 and 7.6 mcg/mL at 3 weeks postpartum, which are at or below the proposed maintenance threshold of >9 mcg/mL.^11^ Despite these relatively low concentrations, the patient remained in sustained clinical remission, indicating adequate disease control.

**FIGURE 1.**
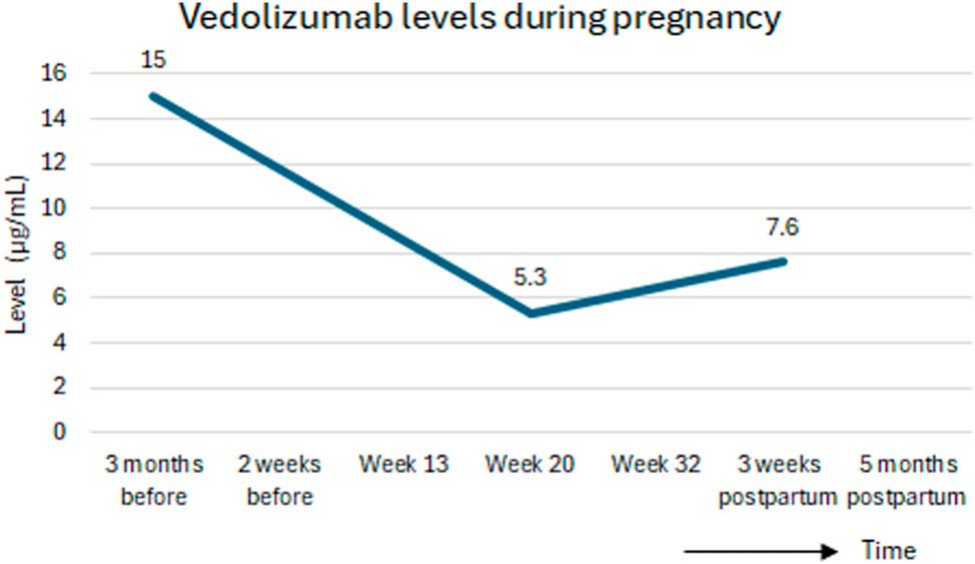
Vedolizumab concentrations during pregnancy.

The infliximab curve does not entirely reflect the increase typically described in literature (Fig. 2).^6,7^ The serum concentration was 38 mcg/mL 2 weeks before pregnancy and rose from 22 mcg/mL to 28 mcg/mL between week 13 and 32 of pregnancy, despite a dose reduction from 10 to 5 mg/kg every 4 weeks starting at approximately week 18. At 5 months postpartum the concentration dropped to 19 mcg/mL without changing the dose. For infliximab, a concentration of >3–7 mcg/mL was considered sufficient for maintenance therapy.^12^ However, in this case, concentrations were consistently well above this therapeutic range, reflecting the patient's individual requirements for maintaining remission. The lower limits of quantification for vedolizumab and infliximab were 100 ng/mL and 0.1 mg/L, respectively. Because the drug concentrations were within the therapeutic range, anti-drug antibodies were not assessed.^1,9^

**FIGURE 2.**
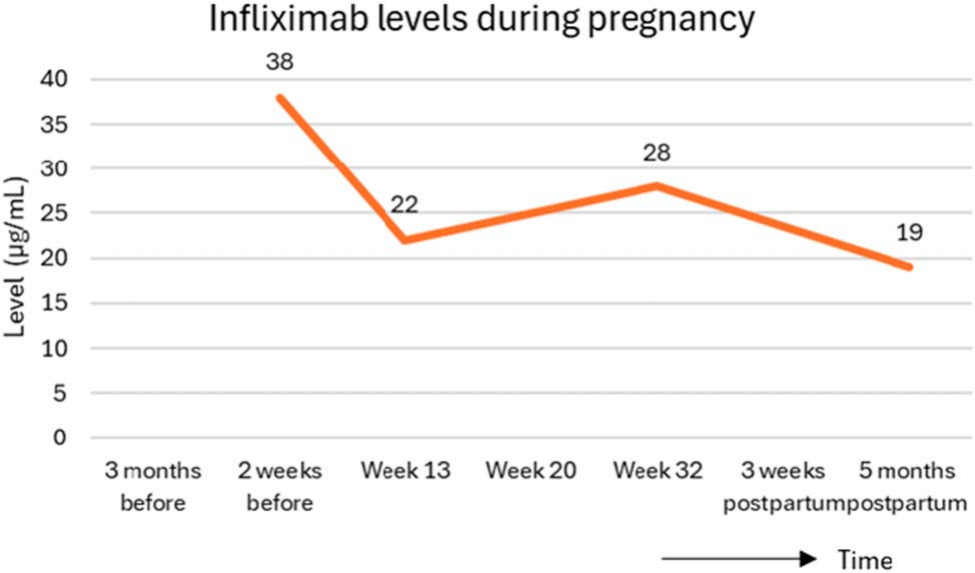
Infliximab concentrations during pregnancy.

## CLINICIAN

The infliximab concentration in the umbilical cord was 41 mcg/mL, higher than the maternal level at 28 mcg/mL, measured 1.5 weeks before delivery. The vedolizumab umbilical cord concentration was 1.0 mcg/mL, which is lower than the last measured maternal concentration. Do these concentrations in the child align with expectations based on existing literature?

## TDM CONSULTANT

Infliximab concentrations in infants can exceed maternal concentrations, reflecting fetal accumulation as pregnancy progresses, consistent with typical IgG1 placental transfer.^13^ By contrast, vedolizumab, also an IgG1 antibody, shows lower infant-than-maternal concentrations, likely due to limited placental transfer, although the mechanism remains unclear. Vedolizumab may remain bound within the placenta rather than being actively transferred to the fetus.^7^ Vedolizumab concentrations in infants are typically undetectable by 16 weeks, with a mean clearance of 10.5 weeks (range 8–16 weeks).^7^ For infliximab, infant clearance after intrauterine exposure averages 7.3 months.^13^

## CLINICIAN

Although both autoimmune diseases remained clinically stable during pregnancy, the infant was delivered prematurely, with a good birth weight of 2425 g (p85), without growth restriction. This aligns with existing evidence that pregnant women with autoimmune conditions are at increased risk of adverse pregnancy outcomes, including preterm birth.^14^ Possible explanations include placental insufficiency or activation of the hypothalamic–pituitary–adrenal (HPA) axis in a growth restricted or otherwise at risk fetus.^15^ In this case, preterm delivery occurred despite well-controlled disease during pregnancy, underscoring the complex interplay between maternal autoimmune disease and pregnancy outcomes. Gestational diabetes and mild polyhydramnios may also have contributed to preterm delivery.^1^

## CONCLUSIONS

A 32-year-old woman with juvenile rheumatoid arthritis and Crohn's disease is presented. After years of therapy with various medications, she was treated with two biologics (infliximab and vedolizumab), continued throughout pregnancy. Therapeutic drug monitoring plays a role in optimizing dosing and effectively managing both conditions, highlighting the need for individualized interpretation. Despite satisfactory disease control, she delivered a preterm but healthy infant. Autoimmune diseases increase the risk of adverse pregnancy outcomes, possibly via placental insufficiency or HPA activation. In addition, gestational diabetes and mild polyhydramnios may have contributed to preterm delivery in this case. Multidisciplinary management—in this case a gastroenterologist, rheumatologist, gynecologist, surgeon, and clinical pharmacist with dedicated expertise in IBD and pregnancy—is essential to ensure comprehensive, high-quality care in pregnant women with multiple autoimmune diseases, particularly given the absence of established guidelines. Future research using a prospective cohort should focus on drug concentrations, disease activity, and risk of adverse pregnancy outcomes, to enhance our understanding of dual biological treatment during pregnancy.
